# Mental health and burnout among Swiss medical students living in contexts of deprivation: a longitudinal study

**DOI:** 10.1136/bmjopen-2025-114025

**Published:** 2026-07-29

**Authors:** Madeline Chardon, Patrick Bodenmann, Valerie Carrard, Alexandre Berney, Véronique S Grazioli

**Affiliations:** 1 Department of Vulnerability and Social Medicine, Center for Primary Care and Public Health, Chair of Medicine for Vulnerable Populations, University of Lausanne, Lausanne, Switzerland; 2 University of Lausanne, Lausanne, Switzerland; 3 Department of Psychiatry, Psychiatric Liaison Service, Lausanne University Hospital (CHUV) and University of Lausanne, Lausanne, Switzerland

**Keywords:** Medical education & training, mental health, public health, health equity, longitudinal studies

## Abstract

**Background:**

The mental health of medical students has been widely discussed, with studies reporting a high prevalence of mental health issues, including depression, anxiety, suicidal ideation and burnout. Yet, few have explored the relationship between material, social and health deprivation and these outcomes in medical students, despite the higher risk of psychological disorders when exposed to precarious living conditions.

**Objective:**

Our study aimed to explore longitudinal associations between experiencing deprivation and mental health outcomes among Swiss medical students while adjusting for protective factors.

**Design:**

Data were collected from hour-long online surveys at two time points (November–December 2022 and November–December 2023) of an open cohort study of medical students from the University of Lausanne, Switzerland (UNIL). Data were analysed using hierarchical multiple regression models.

**Setting:**

This study was carried out by Lausanne University Hospital (Centre Hospitalier Universitaire Vaudois (CHUV)) and the Centre for Primary Care and Public Health, UNIL.

**Participants:**

Our sample included medical students enrolled in any year of medical school studies, excluding those enrolled as part of an exchange programme. At time points T3 and T4, there were, respectively, 1941 and 1267 eligible students for participation; the final sample consisted of 631 medical students having participated at both time points (67.5% female, mean age 22.11).

**Primary outcome measures:**

Deprivation was evaluated with the Deprivation in Primary Care Questionnaire (DIPCare-Q) index. Mental health variables included depression symptoms, anxiety symptoms, suicidal ideations and burnout symptoms (emotional exhaustion, cynicism, academic efficacy). Protective factors included coping skills (emotion-focused, problem-focused and help-seeking) and social support (practical and emotional support).

**Results:**

In our final regression model, controlling for demographics (ie, age, gender, year of study and mother tongue), protective factors and baseline mental health scores, a higher overall baseline deprivation index was predictive of increased depression (β=0.14, p<0.001) and anxiety (β=0.06, p=0.046) symptoms over time, but not for suicidal ideation or burnout symptoms.

**Conclusion:**

Results demonstrated a significant association between deprivation and mental health longitudinally. Findings suggested that social support was protective from an increase in suicidal ideations over time. The implementation of interventions that provide material and social support may help promote improved mental health and health equity among medical students.

**STRENGTHS AND LIMITATIONS OF THIS STUDY**

- A 2-year longitudinal approach was used to assess the relationship between an overall deprivation index and mental health outcomes.
- Results were derived from a large population of Swiss medical students (N=631).
- Incorporating coping styles and social support refines analyses by accounting for the potential confounding effects of these protective factors.
- Possible selective attrition (attrition rate 33.4%) may have led to an underestimation of longitudinal associations between experienced deprivation and mental health, as single-time point participants reported higher deprivation and poorer mental health.

##  Introduction

Epidemiological studies have consistently reported high prevalences of mental health issues among medical students, such as depression, anxiety, suicidal ideation^1–5^ and burnout syndrome.^6^ In 2022, Swiss medical students reported significantly higher depression and anxiety symptoms compared with an age-matched population.^7^ The Social Determinants of Health (SDH) framework outlines the roles of economic, social, political and environmental conditions in health and their impact on health equity.^8 9^ Material and social deprivation (MSDep) describes “a state of observable and demonstrable disadvantage relative to (…) a society”^10 11^ in relation to these conditions.^8 12^

When referring to deprivation, we highlight its ‘relative’ nature, as living in a context of deprivation does not exclusively affect those living in poverty. A person may live in a state of deprivation while exceeding the minimum standard of living if their social conditions fall below the societal average;^10^ this distinction is essential as both financial and social exclusion have been shown to negatively affect mental health.^13^

In 2022, approximately 4.9% of the Swiss population was affected by social and material deprivation.^14^ According to the Swissuniverities organisation^15^ and the University of Lausanne,^16^ the cost of living for a medical student at the University of Lausanne is approximately equivalent to 23 200 CHF per year (approx. US$29 000). This includes tuition fees (1000 CHF or US$1200/year at the University of Lausanne, Switzerland (UNIL)), accommodation (approx. 700 CHF or US$900/month), food, clothing, health insurance (90 CHF or US$120/month) and other expenses (transport, phone bills, etc). These indicators concern material deprivation.

MSDep has been shown to negatively impact mental health among the general population.^13 17–19^ However, research exploring this relationship in medical students remains limited, with few studies examining multiple factors or using longitudinal design. Literature has found difficulty paying bills^20^ and low socioeconomic status^21–25^ to be linked to higher levels of depression, anxiety and suicidal ideation in medical students internationally.^3 26–28^

Among the few studies incorporating multiple markers of MSDep identified, one cross-sectional study of medical students in Czechia, Iran, Kenya and Venezuela associated low socioeconomic status, limited personal budget and reduced social life with higher stress, depression and anxiety,^25^ while another found greater burnout among the most deprived UK medical post-graduates.^29^ When studies examine individual factors, the complex interplay between financial strain and its social and health consequences is overlooked. Furthermore, overall MSDep remains challenging, highlighting the need for effective assessment tools. Recognising this, the DIPCare research team created the Deprivation in Primary Care Questionnaire (DIPCare-Q) score, designed for Swiss primary care settings, to assess material (eg, housing, financial burden, etc), social (isolation, leisure/recreational activities, etc) and health deprivation.^30 31^ To our knowledge, no studies have applied this tool to investigate mental health in any population.

In response to these gaps in data, we aimed to explore the relationship between Swiss medical students’ reported deprivation and mental health outcomes over time, using the DIPCare-Q tool. Additionally, we wished to assess the potential confounding effects of protective factors on this relationship by integrating three coping styles (emotion-focused, problem-focused and help-seeking) and social support. Literature has associated these strategies with lower levels of stress and depression symptoms in medical students.^32 33^ In American and Portuguese medical students, higher levels of social support (ie, receiving emotional, informational and tangible support from others^34^) were related to greater resiliency (ie, never experiencing burnout),^35 36^ whereas lower perceived social support increased the likelihood of depression and burnout symptoms in American and Syrian medical students.^37 38^

Thus, our study aimed to (1) cross-sectionally and longitudinally examine the associations of deprivation and mental health and burnout symptoms among medical students and (2) examine these associations while accounting for protective factors. Based on previous research,^18 24–26 39^ we expected higher experienced deprivation to predict worsening depression, anxiety and suicidal ideation over time. We anticipated similar outcomes regarding burnout symptoms, although this hypothesis is less supported as data regarding this relationship in medical students are limited.^29 40^

## Method

### Design and data collection

This study was a secondary analysis of the Longitudinal Study of Medical Students’ Interpersonal Competence and Mental Health (ETMED-L)^41^ project, funded by the Swiss National Science Foundation (10001C_197442/1) and Grant FBM-Solis financed by the Fondation Solis (in collaboration with the Fondation Centre Hospitalier Universitaire Vaudois (CHUV)) and the Faculty of Biology and Medicine, UNIL. The open cohort study administered hour-long online surveys at four time points between 2021 and 2023 to medical students enrolled at the UNIL, comprised of mental health screening measures and interpersonal competence assessment tools. The study adopted an open-cohort design, allowing new participants to enter the cohort at any measurement point. At every wave, all medical students enrolled at the University of Lausanne (except for external academic exchange students) were invited to participate. Students could take part for the first time or re-enrol at subsequent waves. Note that to prioritise longitudinal continuity, first-year students were not invited at T4. Students were invited to participate by email at each study time point and received 50 CHF (approx. US$50) per completed survey. Although data were also collected at T1 (March 2021) and T2 (November 2021), these waves were not included in the present study because the DIPCare-Q index—central to our focus on deprivation—was introduced only from T3. The present study analysed data collected from evaluations T3 (November–December 2022) and T4 (November–December 2023) of the ETMED-L project.

### Population

In Switzerland, students may enrol in medical school immediately out of their high school years without prior higher-education degrees. Thus, students generally begin studies between the ages of 18 and 20 years old. At the UNIL, the medical school curriculum is comprised of a 3-year bachelor’s degree in human medicine followed by a 3-year master’s degree in human medicine. The bachelor curriculum years (1–3 years) are considered preclinical while master years (4–6) are clinically oriented. Once both degrees are obtained and the Swiss Federal board examinations are passed, students are considered licensed physicians in Switzerland and continue post-graduate training towards a Fédération des Médecins Helvétiques specialist title awarded by the Swiss Institute for Medical Education.

Sample size calculation was conducted by the team of the overall ETMED-L study.^41^ They estimated an enrolment average of 1500 students from first to sixth year per year at the University of Lausanne Medical School. The Monte Carlo method was used to estimate potential statistical power in individuals’ response change between two time points, revealing an expected 77% power in detecting effect size and over 93% in the detection of eventual covariate effect. Inclusion criteria for every study time point required enrolment as a medical student in any year (one through six) at the UNIL. Exclusion criteria included enrolment in the faculty as part of an exchange programme. The ETMED-L study expected a response rate of 35% for each study wave through student compensation. Across the different time points, between 49.3% and 64.6% of eligible students participated, resulting in an average participation rate of 54.6%. At our studied time points T3 and T4, there were respectively 1941 and 1267 eligible students for participation. A flowchart of student participation at each time point is available as online supplemental (participant flowchart).

#### Participant involvement

Although participant input was not used in the design and conduct of this study, we intend to specifically disseminate results to the studied population, namely Swiss medical students from the UNIL.

### Measures

#### Sociodemographic characteristics

Sociodemographic characteristics were measured at T3 (ie, age, gender, year of study and mother tongue). Self-identified gender was determined as male, female or non-binary. Participant nationality was qualified as ‘Swiss’ if a participant chose Swiss-French, Swiss-German or Swiss-Italian as their mother tongue. Selecting another language was coded as ‘other nationality’. Preclinical and clinical stages were not included as a measure as first-year students were not eligible for participation at T4 to prioritise longitudinal follow-up, and sixth-year students at T3 could not be followed up due to graduation. These variables were used to describe the sample and were added as covariates in analyses.

#### Deprivation

The DIPCare-Q questionnaire evaluated overall deprivation.^30 31^ This questionnaire is comprised of 16 items, evaluating material, social and health deprivation with a Yes/No dichotomous scale. Following the equation guideline, an overall deprivation index was calculated. The overall index calculation equation and item examples have been provided as supplemental information (online supplemental appendix A.1).

#### Depression, anxiety, suicidal ideation and burnout

Detailed descriptions of mental health and burnout measures can be found in the ETMED-L protocol.^41^

Depression symptoms were assessed with the Centre for Epidemiological Studies-Depression (CES-D).^42 43^ The final CES-D score was calculated by the mean of its 20 items (⍺=0.91 at T3). Anxiety symptoms were assessed with the ‘Trait’ section of the State-Trait Anxiety Inventory score, through the mean of its 20 items (⍺=0.89 at T3).^44 45^ Suicidal ideation was measured with two questions from the Beck Depression Inventory, which were summed to calculate a total score (Mean Inter-Item Correlations (MIIC)=0.40 at T3).^46 47^ Specific item responses are available (online supplemental appendix A.2).

Burnout was measured with the Maslach Burnout Inventory Student-Survey, a 15-item questionnaire assessing emotional exhaustion (n=5, ⍺=0.86, MIIC=0.55 at T3), cynicism (n=4, ⍺=0.85, MIIC=0.56 at T3) and academic efficacy (n=6, ⍺=0.76, MIIC=0.34 at T3).^48–52^ The latter is considered a reverse dimension of burnout; scoring higher in emotional exhaustion and cynicism subscales and lower in academic efficacy was indicative of burnout.^51^

Depression, anxiety, suicidal ideations and burnout symptoms at T4 were used as outcomes whereas their measures at T3 were added as covariates in the longitudinal analyses.

#### Protective factors

Descriptions of protective factors can be found in the ETMED-L protocol.^41^ Coping strategies were evaluated with a total of 17 items from the Euronet questionnaire,^53–56^ assessing emotion-focused (n=9, ⍺=0.55, MIIC=0.12), problem-focused (n=4, ⍺=0.38, MIIC=0.13) and help-seeking coping (n=4, ⍺=0.61, MIIC=0.29) strategies. Social support was assessed with two questions adapted from the Swiss Household Panel questionnaire (Appendix A.3), evaluating perception of available practical support and emotional support.^57^ Both items were evaluated from 0 to 10 (10=highest amount of support); then, a mean score was calculated from their total sum (⍺=0.75, MIIC=0.60). Protective factors at T3 were used as covariates in analyses.

### Statistical analysis

Outcomes were examined for outliers and deviation from expected distributions; outcomes followed normal distributions. Hence, hierarchical linear regression models were conducted in six steps to assess associations between baseline deprivation at T3 and dependent variables (ie, depression, anxiety, suicidal ideation and burnout scores) both cross-sectionally and longitudinally. Our study focused on the prospective association between deprivation and mental health outcomes rather than modelling deprivation score changes and their association with changes in well-being.

Steps 1 through 3 assessed cross-sectional associations between deprivation and the mental health outcome at T3, with adjustment for demographics in step 2, and adjustment for demographics and protective factors in step 3. Steps 4 through 6 assessed the prospective association between deprivation scores at T3 and mental health outcomes at T4. The step 5 model adjusted for demographics and baseline mental health score at T3. In step 6, the model adjusted for demographics, baseline mental health and protective factor scores at T3. Significance was evaluated at p<0.05. All analyses were conducted using IBM SPSS, V.29.^58^

## Results

### Sample

At T3, 1101 (56.7% response rate) eligible students completed the survey; 35 were excluded for failure of attention questions or for technical issues. Analyses required the exclusion of sixth-year students (n=118), as they would be ineligible for participation at T4, leading to a final sample of 948 students. At T4, first-year students were ineligible to participate due to funding restrictions and were chosen for exclusion as their participation would not provide longitudinal data. Among eligible students (n=1267), 849 (67% response rate) completed the survey. The total sample resulted in 819 students after exclusion of questionnaires for failure of attention questions or incoherent answers. A final sample of N=948 students participated at T3. Of these, 631 (66.6%) also participated at T4 while 317 (33.4%) did not, corresponding to an attrition rate of 33.4%. The final sample for longitudinal analyses therefore comprised of 631 students who participated at both T3 and T4.

Independent t-tests and χ^2^ tests were conducted to compare participants who completed both T3 and T4 with those who dropped out after T3. χ^2^ tests indicated a significantly higher longitudinal participation rate among students of ‘Swiss nationality’. There was a significant difference in curriculum year, with single-time point participants being primarily in their first years of study and longitudinal participants in later years. Mean age was significantly higher in the longitudinal participation group. Single-time point participants reported higher overall deprivation indexes, higher depression symptoms, anxiety symptoms, suicidal ideations and academic efficacy. Baseline mental health score was included in step 5 and step 6 to account for attrition variability of these scores detected through listwise deletion. Complete χ^2^ and t-test tables are available as (online supplemental tables S1 and S2).

### Descriptive statistics

Among the 631 double participants at T3, 67.5% identified themselves as female, 32% as male and 0.5% as non-binary. Mean age was 22.11 (SD=2.95) and 78.8% of participants were of ‘Swiss nationality’. Regarding year of study, 13.3% (n=84) of students were enrolled in the first year, 20.8% (n=131) in the second year, 23.6% (n=149) in the third year, 22.7% (n=143) in the fourth year and 9.7% (n=124) in the fifth year. Results of descriptive statistics are presented in table 1.

**Table 1:** Descriptive statistics of demographics, deprivation indexes, protective factors and mental health and burnout scores (N=631)

	% missing	N (%)	M	Mdn	SD	Min	Max
Demographics (T3)							
Age	0.5	628	22.11	—	2.95	17	51
Gender identity	0	631	—	—	—	—	—
Male	—	202 (32)	—	—	—	—	—
Female	—	426 (67.5)	—	—	—	—	—
Non-binary	—	3 (0.5)	—	—	—	—	—
Nationality	0	631	—	—	—	—	—
Swiss*	—	497 (78.8)	—	—	—	—	—
Deprivation in Primary Care Questionnaire (DIPCare-Q)							
Overall deprivation index (index range: 0–4)							
T3	0	631	0.69	1.00	0.81	0	4
Protective factors							
Emotion-focused coping (index range: 0–27)							
T3	0.5	628	9.52	9.00	3.76	0	21
Problem-focused coping (index range: 0–12)							
T3	0.5	628	7.22	7.00	1.73	0	12
Help-seeking coping (index range: 0–12)							
T3	0.5	628	5.58	6.00	2.75	0	12
Social support (index range: 0–10)							
T3	0.5	628	8.19	8.50	1.78	0	10
Mental health							
Depression symptoms (index range: 0–60)							
T3	0.3	629	17.93	17.00	10.35	0	52
T4	0.8	626	17.74	16.00	11.05	0	54
Anxiety symptoms (index range: 20–80)							
T3	0.3	629	45.59	45.00	11.24	20	76
T4	0.8	626	44.86	45.00	11.66	20	77
Suicidal ideation (index range: 0–6)							
T3	0.3	629	0.59	0.00	1.01	0	6
T4	0.8	626	0.58	0.00	0.98	0	6
Burnout							
Emotional exhaustion (index range: 5–30)							
T3	0.5	628	16.66	16.00	4.96	5	30
T4	0.8	626	16.29	16.00	4.88	5	30
Cynicism (index range: 4–24)							
T3	0.5	628	9.16	8.00	4.22	4	24
T4	0.8	626	9.78	9.00	4.25	4	24
Academic efficacy (index range: 6–36)							
T3	0.5	628	24.38	25.00	4.34	6	36
T4	0.8	626	24.05	24.00	4.41	9	36

* Swiss if participant selected mother tongue=Swiss-French, Swiss-German or Swiss-Italian

### Hierarchical linear regression models

Results of regression analyses are available as online supplemental table S3 (cross-sectional) and online supplemental table S4 (longitudinal).

#### Cross-sectional analyses (steps 1–3)

*Step 1*. Analyses testing DIPCare-Q score and mental health outcome associations at T3 revealed significantly positive associations for all dependent variables. *Step 2*. When adjusting for sociodemographic factors, the effect of deprivation remained significant. *Step 3*. When adjusting for protective factors and demographics, deprivation remained significantly associated with increased depression symptoms, anxiety symptoms, suicidal ideation and two burnout symptoms (emotional exhaustion and cynicism; not academic efficacy).

#### Longitudinal analyses (steps 4–6)

*Step 4*. A high deprivation score at T3 was predictive of increased levels of depression symptoms, anxiety symptoms, suicidal ideation, emotional exhaustion and cynicism at T4; this was not significant for academic efficacy. *Step 5*. While considering demographics and baseline T3 score of dependent variables, a higher deprivation index at T3 was predictive of increased depression symptoms, anxiety symptoms and suicidal ideations over time. *Step 6*. When adjusting for demographics, baseline T3 dependent variable scores and protective factors, deprivation at T3 was significantly associated with depression and anxiety symptoms.

## Discussion

### Effect of deprivation on mental health and burnout symptoms

As expected, living in a context of deprivation was significantly associated with increased depression symptoms and anxiety symptoms both cross-sectionally and longitudinally. These results are consistent with the literature^6 25 59 60^ and expanded on previous findings by assessing experienced MSDep, comprehensively relating material and social indicators with mental health. This is in line with the SDH framework,^8 61^ suggesting that MSDep worsens medical students’ mental health and may contribute to outcomes worsening over time. These findings underscore the potential structural changes and protective systems needed to reduce deprivation and support medical students’ mental health throughout their education.

Consistent with previous cross-sectional research,^29 40 62^ findings revealed a positive association between deprivation and concurrent burnout symptoms. This association lost significance when assessed longitudinally, further clarifying this relationship. Compared with mood disorders like depression,^63 64^ caused by endogenous^65 66^ and exogenous factors,^67 68^ burnout syndrome is considered a ‘work-related phenomena’,^49 69–71^ inherently shaped by one’s work or study environment.^72 73^ It may be that the long-term impact of deprivation on burnout syndrome is less pronounced compared with work/study environmental factors given its ‘contextual’ nature. Though ‘transiently’, deprivation may contribute to these difficulties, as shown by our cross-sectional analyses. Given the high prevalence of burnout among medical students (37.23%^74^) and its association with an increased risk of suicidal ideation,^75 76^ addressing deprivation may be an important consideration to avoid such outcomes.

### Protective factors against mental health and burnout symptoms

Our final model adjusted for coping strategies and social support. Regardless of this, the association of deprivation with depression and anxiety symptoms remained significant, supporting our conclusions on the weight of SDH on mental health. Interestingly, the association between deprivation and suicidal ideation lost significance after adjustment, suggesting that social support may serve as a buffer against the risks associated with deprivation. Our findings are consistent with a previous study conducted among Italian medical students, finding that good familial cohesion and being in a relationship were negatively associated with suicidal ideation.^23^ Following Joiner’s Interpersonal-Psychological Theory of Suicidal Behaviour,^77 78^ having a ‘desire of death’ is driven by a sense of ‘low belonging’ or ‘social alienation’ in addition to ‘perceived burdensomeness’.^79 80^ In this context, social support may play a protective role against suicidal ideations by potentially mitigating these cognitive processes. Thus, actions promoting social support across educational institutions may reduce mental health issues, particularly among those living in contexts of MSDep.

### Next steps

#### Interventions to reduce the impact of deprivation

In light of our findings and following the SDH conceptual framework,^8 61^ systemic measures that prioritise social support, financial aid, health and well-being may be beneficial to promoting health equity among students.

Schools and governmental institutions could look into financial measures to expand grants and scholarships to cover educational costs and basic necessities for those in need.^81 82^ Financial advisors, accommodation support, food banks,^82 83^ as well as emergency financial support and accommodation^84 85^ by universities may be a means of providing a safety net for students facing unprecedented difficulties. Faculty training to understand such students’ needs could aid in referring them towards appropriate resources.^82 86^

Individual mental health interventions, notably when combined with educational interventions and staff training, have been proven effective against depression, anxiety and suicidal ideations in medical students.^87–89^ The provision of on-campus psychological counselling at reduced or no cost could be a valuable answer to their needs.^84 90^ Interventions could include in-class seminars on well-being within medical curricula;^87 91^ implementation studies of mindfulness programmes in international medical curricula have revealed significant improvement in depression symptoms.^92–94^ Regarding student outreach, student mental health ambassadors could promote well-being among peers,^84 95^ while staff training and faculty-student mentorships may support early recognition and guidance of students in need.^87 95 96^

#### Reinforcement of protective factors

Our findings suggest that strengthening social support may represent a promising buffer against suicidal ideations in medical students; potential interventions may include individual and group-based approaches.^97^

One-on-one support programmes could include in-person meetings with friends, family members, trained peers or professionals,^97^ whereas online outreach via email or other platforms may offer anonymous support.^97 98^ Group support programmes were shown to decrease suicide-related symptoms,^99^ with peer-led programmes for suicide prevention allowing participants to ‘speak freely’ and ‘increased their sense of community’.^100 101^ Studies show the overall positive impact of online peer-based forums and groups on depression and anxiety in participants,^102 103^ and allowed them to relate with people with similar experiences of suicidal ideations, without ‘burdening’ their loved ones.^104^ Such platforms could be integrated by universities to strengthen social cohesion.

#### Furthering research

Further steps may be taken to gain a deeper understanding of the specific needs that most affect medical students. These may include qualitative studies involving students with increased DIPCare-Q scores to explore their experiences, including the strengths and limitations of university aid regarding deprivation and mental health. These could inform the potential implementation of specific interventions and their subsequent evaluation in response to identified needs. Finally, future studies could explore dynamic changes in deprivation and their relationship with well-being over time.

## Limitations

Certain limitations to our study should be acknowledged. Given the significant drop-out rate of single-time point participants reporting higher deprivation indexes and mental health scores compared with longitudinal participants, these differences may have caused selection bias. We hope to have accounted for this by adjusting for baseline mental health scores. Response bias may have arisen due to inconsistent use of time frames across mental health, deprivation and protective factor measures. Longer or unspecified time frames may have led to under-reporting of symptoms, while shorter time frames may have caused over-reporting, thus affecting the accuracy of the findings. Switzerland has a relatively high economic standing; extrapolating our findings to countries with different economic contexts may be challenging. Additionally, our participants may have differing perceptions of socioeconomic status given their geopolitical context, further limiting the generalisability of our findings.

## Conclusion

Our study explored the effects of experiencing deprivation on medical students’ mental health. Longitudinally, depression and anxiety symptoms were shown to worsen when students reported higher levels of deprivation, regardless of adjustment for protective factors. While these results remain concerning, our findings suggest that social support may be protective against an increase in suicidal ideations over time. Structural measures taken by universities and governmental instances may represent a promising approach to ensuring that students have their basic needs met to support their mental well-being. Access to financial support, social support and mental health resources may play a potentially beneficial role in providing more equal academic opportunities and health equity among medical students.

## Supplementary material

10.1136/bmjopen-2025-114025 online supplemental file 1

10.1136/bmjopen-2025-114025 online supplemental file 2

## Data Availability

Data are available in a public, open access repository.
