# Supplementary material for: Mental health and burnout among Swiss medical students living in contexts of deprivation: a longitudinal study
Source: BMJ Open. 2026 Jul 29;16(7):e114025. doi: 10.1136/bmjopen-2025-114025 (PMC13435915; doi:10.1136/bmjopen-2025-114025)
Supplement: Supplementary file 1 [file bmjopen-16-7-s001.pdf]

Flow chart longitudinal analyses

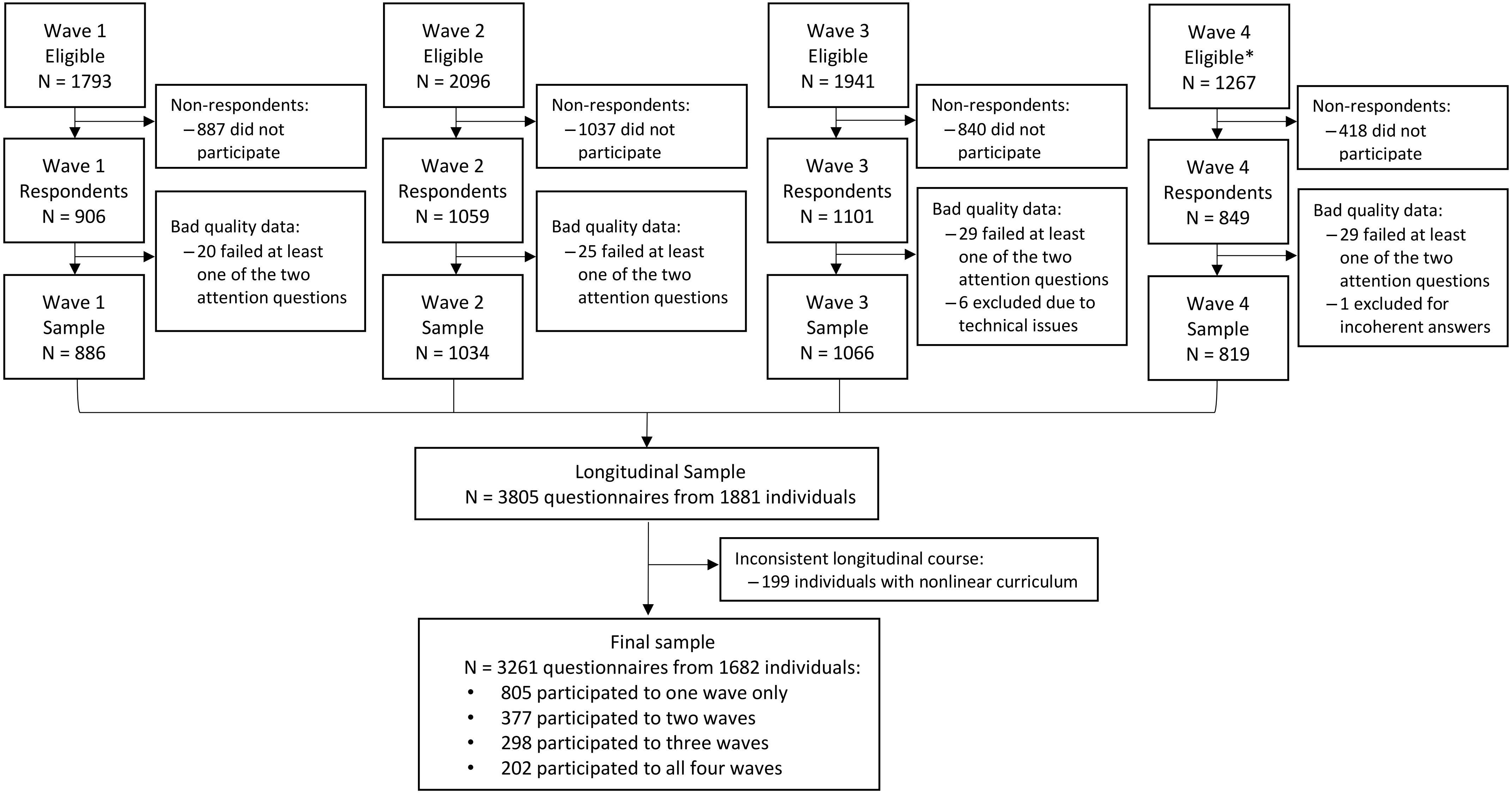

*excluding first year students
