# Supplementary material for: Mental health and burnout among Swiss medical students living in contexts of deprivation: a longitudinal study
Source: BMJ Open. 2026 Jul 29;16(7):e114025. doi: 10.1136/bmjopen-2025-114025 (PMC13435915; doi:10.1136/bmjopen-2025-114025)
Supplement: Supplementary file 2 [file bmjopen-2025-114025.reviewer_comments.pdf]

PEER REVIEW HISTORY

BMJ Open publishes all reviews undertaken for accepted manuscripts. Reviewers are asked to complete a checklist review form and are provided with free text boxes to elaborate on their assessment. These free text comments are reproduced below.

ARTICLE DETAILS

Title (Provisional)

MENTAL HEALTH AND BURNOUT AMONG SWISS MEDICAL STUDENTS LIVING IN CONTEXTS OF DEPRIVATION: A LONGITUDINAL STUDY

Authors

Chardon, Madeline; Bodenmann, Patrick; Carrard, Valerie; Berney, Alexandre; Grazioli, Véronique

---

VERSION 1 - REVIEW

**Reviewer**	**1**
**Name**	**Nash, Carol**
**Affiliation**	**University of Toronto Temerty Faculty of Medicine, Psychiatry**
**Date**	**28-Nov-2025**
**COI**	**None**

An 65-page submission composed of several parts (1) pages 3–36, the submission (reviewed), (2) pages 37–42, a shorter summary of the findings in English (not reviewed), (3) pages 43–48, the same summary in French (not reviewed), (4) pages 49–50, a letter from the corresponding author, including references (not reviewed), (5) pages 51–60 a previously published article of a study protocol for the project (not reviewed), and pages 61–65, the supplemental information for the submission (reviewed).

Exploring experiences of deprivation and mental health outcomes while adjusting for protective factors in 631 Lausanne, Switzerland, medical students, was the aim of this 2022–2023 longitudinal study.

The strengths are (1) the research is original, (2) it follows on the results of previous similar publications by this research team, (3) there are a large number of participants, (4) the length of time was sufficient to see longitudinal results, (5) the methodology is explained in sufficient detail for study replication, (6) the range of the analysis, (7) the conclusion follows from the results, and (8) the completed STROBE 2007 (v4) checklist is included.

The weakness is that there are too many outdated citations.

Page-by-page suggested edits

These suggested edits concern the page and citation numbers of outdated references. For each, please find a supporting citation (do not delete the current one) of research published since 2021. For methodologies, the aim is to demonstrate that the cited one is still considered relevant in the field. The page numbers used are those of the entire document. They are two greater than the top right corner.

8 [1,2], [7], [5], [13,14],[13], and [20–22].

9 [24], [28], and [29].

11 [24], and [33].

12 [34], [35–38], [38], [39–41], and [42].

20 [48], [49], [50], and [36,51].

21 [55], [56], and [57].

22 [58], [59], 62], and [68].

23 [73], and [74].

**Reviewer**	**2**
**Name**	**Popescu, Codruta**
**Affiliation**	**Universitatea de Medicina si Farmacie Iuliu Hatieganu**
**Date**	**23-Jan-2026**
**COI**	**None**

The authors described the inclusion and exclusion criteria, and the number of students who complete the questionnaire at each step, but the manuscript would benefit from a brief clarification regarding the determination of the sample size. Although the final sample is substantial and appropriate for the analyses conducted, it is not specified whether an initial sample size calculation was performed.

In the background it will be interesting to do a contextualization of deprivation within the Swiss medical education setting. Given that deprivation is a central construct in the study, the manuscript would benefit from additional contextual information regarding the economic realities of medical students in Switzerland. Briefly describing the costs associated with medical studies and student living expenses (e.g., tuition, housing, health insurance, cost of living) would help readers better interpret the meaning of material and social deprivation in this high-income setting. The authors may also wish to clarify whether deprivation in this context reflects relative rather than absolute deprivation, and how this distinction may relate to mental health outcomes.

**Reviewer**	**3**
**Name**	**Merlo, Lisa**
**Affiliation**	**University of Florida, Psychiatry**
**Date**	**28-Jan-2026**
**COI**	**None**

Thank you for the opportunity to review, “Mental health and burnout among Swiss medical students living in contexts of deprivation: A longitudinal study.” This is an interesting topic that is not well-studied, but reflects an important area of inquiry related to medical student wellbeing. It would likely be of interest to readers. However, there are some concerns that dampen enthusiasm for the manuscript in its current form. Some recommendations are offered for consideration below.

Abstract:

• Please include more information regarding the study participants

• Please update the Abstract, as needed, after editing the manuscript according to reviewer feedback

Summary:

• Please review the second bullet point for clarity

• The description of the attrition rate as reflecting single timepoint participants with higher deprivation is not consistent with the description in the text

• Given that the DIPCare-Q is an index, and not a scale, the concern about the relatively low Cronbach’s alpha is less relevant. Thus, it probably need not be mentioned in the summary

Introduction:

• In general, the introduction is well-written and includes appropriate references

• There are a few instances of missing words throughout the introduction (and other parts of the manuscript). It would benefit from a careful review.

Methods:

Population

• In general, more clarification is needed, particularly related to the study population and sample of participants.

• What is the structure of the medical school curriculum across the 6 years? Do certain years emphasize classroom (preclinical) vs. clinical learning?

• What is the typical age of medical school matriculation in Switzerland? It is noteworthy that at least one participant was 17 years old, and the average age was 22 years. Given the focus on deprivation experiences, the relatively young age of the sample is important. Do most medical students live independently? With roommates? With parents?

• This study appears to include data from time 3 (T3) and time 4 (T4) of a larger study. At what point were the participants recruited? At T1 or on a rolling basis across all time points?

• When did T1 and T2 occur? What data were collected at T1 and T2? Why aren't those data included in this study?

• The inclusion criterion was enrollment in any year of medical school—was that at T1? The text appears to offer contradicting information related to this in different parts (e.g., supplemental tables).

• Why were so many fewer students eligible for participation at T4 compared to T3? Were students who did not participate at earlier timepoints eligible to participate in later timepoints?

• How many total students were eligible to participate at T1? What was the response rate from the total eligible number of students? (i.e., how many medical students attend the medical school and were invited to participate?)

Measures

• The deprivation measure seems to ask about a different time frame for each sample item (e.g., past 12 months, past 3 months, currently). Do the other items vary in this way? How might this discrepancy affect the study results?

• Was the DIPCare-Q completed at T3 and T4? If so, why were T3 scores used to predict T4 mental health results (rather than T4 scores, which corresponded to deprivation experiences between T3 and T4)?

• It is unclear why year in school was not included as a specific predictor or covariate, particularly given that school year (i.e., preclinical vs. clinical phase) has been identified as a significant predictor of medical student wellbeing in multiple studies.

Results:

• The calculations of response rates are confusing. For example, 1101 respondents out of 1941 eligible participants would be 56.7%; 849 out of 1267 eligible participants would be 67%.

• The text-based description of how many individuals participated is inconsistent with the information in supplemental Table 1. Please clarify.

• It may be preferable to use a different term (other than "single participants") to describe individuals who only provided data at one timepoint, in order to prevent confusion with unmarried participants.

• Do the authors have any hypotheses/explanation for why participants in their first year were less likely to participate at both time points? Were the single timepoint participants the individuals who were not eligible to participate at T4?

• In section 3.2, the results seem to reflect only the double timepoint participants—for example, n=84 students are described as being in their first year, but in the supplemental table 1, it indicates that n=190 students participated. Please clarify.

• Please consider conducting analyses to assess whether there is a significant association between year or preclinical/clinical phase of school and wellbeing.

• My primary concerns relate to the longitudinal analyses—for step 4, please explain the rationale for considering deprivation only at T3, and not T4 (or not change in deprivation from T3 to T4) when predicting mental health at T4. For steps 5 and 6, please clarify whether deprivation at T3 or T4 is included in the analysis.

• Consider conducting additional analyses to explore whether change in deprivation status is associated with change in wellbeing measures. Given that the DIPCare-Q assesses deprivation in the recent past, it may be a better measure to assess current wellbeing. (It would be preferable to control for year in school or at least preclinical vs. clinical phase when conducting these analyses.)

Discussion

• The Discussion will likely require significant revision after edits are made to the results section.

• The discussion appears to draw conclusions and make assertions that extend beyond the study results. Please temper these assertions to ensure accuracy.

• Additional limitations include use of self-report data and associated risks for biased reporting, unclear generalizability give the confusing response rates, variability in time frame for the deprivation questions (making it difficult to draw conclusions about direct impact, lack of information about deprivation experiences in early life, unequal representation of class years in the sample.

VERSION 1 - AUTHOR RESPONSE

Reviewer: 1

1. The weakness is that there are too many outdated citations.
Page-by-page suggested edits
These suggested edits concern the page and citation numbers of outdated references. For each, please find a supporting citation (do not delete the current one) of research published since 2021. For methodologies, the aim is to demonstrate that the cited one is still considered relevant in the field. The page numbers used are those of the entire document. They are two greater than the top right corner.
8 [1,2], [7], [5], [13,14],[13], and [20–22].

9 [24], [28], and [29].
11 [24], and [33].
12 [34], [35–38], [38], [39–41], and [42].
20 [48], [49], [50], and [36,51].
21 [55], [56], and [57].
22 [58], [59], 62], and [68].
23 [73], and [74].

Thank you for this comment. Indeed, a notable number of citations used in our paper were published prior to 2021. As asked, new references to compliment those used have been added for each citation mentioned with certain modifications to the text as cited below.

For references 13 and 14, we were unable to find more up to date articles (both from Chinese and international researchers) supporting our text. Thus, we have taken the liberty to modify the text to include only up-to-date references, while retaining reference 13 (now reference 24) given its continuitiy with other cited literature . The text has been modified as follows (page 6-7):

Literature has found difficulty paying bills [20] and low socio-economic status [21–25] to be linked to higher levels of depression, anxiety, and suicidal ideation in medical students internationally [3,26–28].

For references 28 and 29, we have found additional supporting references and have modified the text as follows (page 7-8):

In American and Portuguese medical students, higher levels of social support (i.e., receiving emotional, informational and tangible support from others [34]) were related to greater resiliency (i.e. never experiencing burnout) [35,36], whereas lower perceived social support increased the likelihood of depression and burnout symptoms in American and Syrian medical students [37,38].

Pertaining to our deprivation (DIPCare-Q) mental health (CES-D, STAI, MBI-SS, BDI) and coping strategy (Euronet questionnaire) measures as well as Joiner ‘s Interpersonal-Psychological Theory of Suicidal Behavior, we have added references of studies from 2021 and on that have used these same measures to demonstrate that their relevance has been maintained in the academic community.

Page 11 of manuscript:
- DIPCare-Q: previous reference 24, added reference 31 (article published 2022)
- CESD: no previous reference, added reference 42 and 43 (article published 2021)
- STAI: previous reference 33, added reference 45 (article published 2021)
- BDI: previous reference 34, added reference 47 (article published 2022)
- MBI-SS: previous references 35-38, added reference 52 (article published 2022)
- Euronet coping strategies: previous references 39-41. To our knowledge, the most recent study using the French version of the Euronet coping strategies was published in 2017. We acknowledge that this measure may not the most up to date. However, we retained this instrument as it was considered appropriate by our ETMED-L colleagues at the time of the overall study design in 2020, and its questionnaire items have been used in similar studies conducted in Switzerland. Thus, we have cited this supporting reference as 56.

Page 21 of manuscript:
- Joiner ‘s Interpersonal-Psychological Theory of Suicidal Behaviour: previous references 56 and 57, added references 78 (article published 2021) and 80 (article published 2024) respectively

For reference 68, we have found additional supporting references from internation studies and have modified the text as follows (page 22):
Interventions could include in-class seminars on wellbeing within medical curricula [87,91]; implementation studies of mindfulness programmes in international medical curricula have revealed significant improvement in depression symptoms [92–94].

Reviewer: 2

1. The authors described the inclusion and exclusion criteria, and the number of students who complete the questionnaire at each step, but the manuscript would benefit from a brief clarification regarding the determination of the sample size. Although the final sample is substantial and appropriate for the analyses conducted, it is not specified whether an initial sample size calculation was performed.

Thank you for this valuable comment. In response, we have added clarification regarding the determination of sample size as follows (page 9):

Sample size calculation was conducted by the team of the overall ETMED-L study (32). They estimated an enrolment average of 1500 students from 1st to 6th year per year at the University of Lausanne Medical school. The Monte Carlo method was used to estimate potential statistical power in individuals’ response change between two time points, revealing an expected 77% power in detecting effect size and over 93% in the detection of eventual covariate effect.

2. In the background it will be interesting to do a contextualization of deprivation within the Swiss medical education setting. Given that deprivation is a central construct in the study, the manuscript would benefit from additional contextual information regarding the economic realities of medical students in Switzerland. Briefly describing the costs associated with medical studies and student living expenses (e.g., tuition, housing, health insurance, cost of living) would help readers better interpret the meaning of material and social deprivation in this high-income setting.

We would like to thank the editor for this interesting point they’ve raised. Indeed, to provide additional context to our study sample, we’ve added the following information on page 6:
According to the Swissuniverities organisation [15] and the University of Lausanne [16], the cost of living for a medical student at the University of Lausanne is approximately equivalent to 23’200 CHF per year (approx. 29’000USD) . This includes tuition fees (1000CHF or 1200USD/year at UNIL), accommodation (approx. 700CHF or 900USD/month), food, clothing, health insurance (90CHF or 120USD/month) and other expenses (transport, phone bills, etc). There indicators concern material deprivation.

3. The authors may also wish to clarify whether deprivation in this context reflects relative rather than absolute deprivation, and how this distinction may relate to mental health outcomes.

To address this comment, we have added the following statement to page 6:
When referring to deprivation, we highlight its “relative” nature, as living in a context of deprivation does not exclusively affect those living in poverty. A person may live in a state of deprivation while exceeding the minimum standard of living if their social conditions fall below the societal average [10]; this distinction is essential as both financial and social exclusion have been shown to negatively affect mental health [13].

Reviewer: 3

1. Abstract:
1.1. Please include more information regarding the study participants

We would like to thank the reviewer for this suggestion We have added the following information the abstract (page 3):

Our sample included medical students enrolled in any year of medical school studies, excluding those enrolled as part of an exchange programme. At time points T3 and T4, there were respectively 1941 and 1267 eligible students for participation; the final sample consisted of 631 medical students having participated at both time points (67.5% female, mean age 22.11).

1.2. Please update the Abstract, as needed, after editing the manuscript according to reviewer feedback

As per this suggestion, the abstract has been modified according to reviewer feedback (page 3-4).

2. Summary:
2.1. Please review the second bullet point for clarity

As per this suggestion and the previous made by other reviewers, the summary has been modified for clarity and in alignment with summary guidelines (page 5).

2.2. The description of the attrition rate as reflecting single timepoint participants with higher deprivation is not consistent with the description in the text

We thank you for having noticed this inconsistency. We have modified the summary point as follows (page 5):

• Possible selective attrition (attrition rate 33.4%) may have led to an underestimation of longitudinal associations between experienced deprivation and mental health, as single-timepoint participants reported higher deprivation and poorer mental health.
2.3. Given that the DIPCare-Q is an index, and not a scale, the concern about the relatively low Cronbach’s alpha is less relevant. Thus, it probably need not be mentioned in the summary

We thank the reviewer for this valuable comment. In response, we have removed the summary point (page 5), the Cronbach’s alpha value in the Methods (page 10) as well as in the limitations section (page 23) of the manuscript.

3. Introduction: In general, the introduction is well-written and includes appropriate references. There are a few instances of missing words throughout the introduction (and other parts of the manuscript). It would benefit from a careful review.

Thank you for this kind feedback. In response, the manuscript has been meticulously reviewed for clarity and any missing information.

4. Methods:
4.1. Population
4.1.1. In general, more clarification is needed, particularly related to the study population and sample of participants.

Thank you for this comment. We have added the following information to clarify our population and sample as follows (page 9-10):

Inclusion criteria for every study time point required enrolment as a medical student in any year (one through six) at the UNIL. Exclusion criteria included enrolment in the faculty as part of an exchange programme. At our studied time points T3 and T4, there were respectively 1941 and 1267 eligible students for participation The ETMED-L study expected a response rate of 35% for each study wave through student compensation.

4.1.2. What is the structure of the medical school curriculum across the 6 years? Do certain years emphasize classroom (preclinical) vs. clinical learning?

We thank the reviewer for these pertinent questions. To clarify the local medical curriculum, we have added the following information on page 9:

At the UNIL, the medical school curriculum is comprised of a three-year bachelor's degree in human medicine followed by a three-year master's degree in human medicine. The Bachelor curriculum years (1-3 year) are considered preclinical while Master years (4-6) are clinical oriented. Once both degrees are obtained and have passed the Swiss Federal board examinations, students are considered licensed physicians in Switzerland and continue post-graduate training towards an FMH (Fédération des Médecins Helvétiques) specialist title awarded by the Swiss Institute for Medical Education (ISFM).

4.1.3. What is the typical age of medical school matriculation in Switzerland? It is noteworthy that at least one participant was 17 years old, and the average age was 22 years. Given the focus on deprivation experiences, the relatively young age of the sample is important. Do most medical students live independently? With roommates? With parents?

Thank you for these questions. We have added the following information for further description of our study population (page 9):

In Switzerland, students may enroll in medical school immediately out of their high school years without prior higher-education degrees. Thus, students generally begin studies between the ages of 18-20 years old.

4.1.4. This study appears to include data from time 3 (T3) and time 4 (T4) of a larger study. At what point were the participants recruited? At T1 or on a rolling basis across all time points?

Thank you for this comment. The larger study used an open-cohort design, with recruitment occurring at each data collection point. At every wave, an invitation was sent to all medical students enrolled at the University of Lausanne, excluding external academic exchange students. Each eligible student was free to enrol for the first, second, third, or fourth time at each data collection point. However, note that at the final data collection (T4), first year students were not eligible, as the project prioritized longitudinal follow-up at that stage (see also our answer to your next comments). For greater clarity, the description of the design has been enriched as follows (page 8):
The study adopted an open-cohort design, allowing new participants to enter the cohort at any measurement point. At every wave, all medical students enrolled at the University of Lausanne (except for external academic exchange students) were invited to participate. Students could take part for the first time or re-enrol at subsequent waves. Note that to prioritize longitudinal continuity, first-year students were not invited at T4.

4.1.5. When did T1 and T2 occur? What data were collected at T1 and T2? Why aren't those data included in this study?

T1 took place in March 2021 and T2 in November 2021. The questionnaires used at T1 and T2 were identical to those administered at T3 and T4, with one exception: the DIPCare Q was not yet included and was introduced only from T3 onward. Because the present study focuses specifically on deprivation, the data collected at T1 and T2 were not included in the analyses. To clarify this, the following sentence has been added in the manuscript on page 9:

Although data were also collected at T1 (March 2021) and T2 (November 2021), these waves were not included in the present study because the DIPCare Q index —central to our focus on deprivation—was introduced only from T3. The present study analysed data collected from evaluations T3 (Nov.-Dec. 2022) and T4 (Nov.-Dec. 2023) of the ETMED-L project.

4.1.6. The inclusion criterion was enrolment in any year of medical school—was that at T1? The text appears to offer contradicting information related to this in different parts (e.g., supplemental tables).

As mentioned in point 4.1.4., students of every curriculum year were free to enrol at each data collection point except at the final data collection (T4), where first year students were not eligible to prioritise longitudinal follow-up. To avoid any confusion for readers, this precision in inclusion criteria and participation has been added to the manuscript (page 9-10) per point 4.1.1.

4.1.7. Why were so many fewer students eligible for participation at T4 compared to T3? Were students who did not participate at earlier timepoints eligible to participate in later timepoints?

Thank you for this comment. The inclusion criteria were identical across all measurement points except the final one. At each wave, all medical students enrolled at the University of Lausanne—excluding external academic exchange students—were eligible to participate. At the last data collection point (T4), first-year students were additionally excluded to prioritize longitudinal follow up. This explains why fewer students were eligible at T4. We have added additional information to the manuscript on page 8 and 10 to clarify this point.

4.1.8. How many total students were eligible to participate at T1? What was the response rate from the total eligible number of students? (i.e., how many medical students attend the medical school and were invited to participate?)

Because enrolment occurred at each data collection point, the number of eligible students varied depending on the number of students enrolled in the University at the time. A total of 1793, 2096, 1941, and 1267 students were eligible at T1, T2, T3, and T4, respectively. As you correctly noted, eligibility at T4 was substantially lower because first-year students were excluded at that wave. Across the different time points, between 49.3% and 64.6% of eligible students participated, resulting in an average participation rate of 54.6%. We have added the following information to clarify this point which can be found on pages 9-10:

The ETMED-L study expected a response rate of 35% for each study wave through student compensation. Across the different time points, between 49.3% and 64.6% of eligible students participated, resulting in an average participation rate of 54.6%. At our studied time points T3 and T4, there were respectively 1941 and 1267 eligible students for participation.

4.2. Measures
4.2.1. The deprivation measure seems to ask about a different time frame for each sample item (e.g., past 12 months, past 3 months, currently). Do the other items vary in this way? How might this discrepancy affect the study results?

Thank you for raising this valuable point. Indeed, among our mental health, protective factors and deprivation index, different time frames are assessed for these items. Indeed, the DIPCare-Q index uses a 12-month, 3-month and current time point depending on the item. The CES-D screens for experienced depression symptoms in the past week, while the STAI evaluates present feelings of anxiety as well as generalised feelings in their day-to-day life. The BDI assesses suicidal ideation and pessimism over the last week. MBI-SS screens for generalised burnout symptoms, without a specific timeframe. Neither the Swiss Household Panel social support items nor the coping strategy items from Euronet use specific timeframes. The discrepancy of the use of a time frame and its duration may have caused response bias (e.g. a shorter time frame may lead to better recall of symptoms compared to longer time frames). In response to this comment, we have added the following sentence to the limitations section of the manuscript (page 23):

Response bias may have arisen due to inconsistent use of time frames across the mental health, deprivation and protective factor measures. Longer or unspecified time frames may have led to under-reporting of symptoms, whereas shorter time frames may have caused over-reporting, thus affecting the accuracy of the findings.

4.2.2. Was the DIPCare-Q completed at T3 and T4? If so, why were T3 scores used to predict T4 mental health results (rather than T4 scores, which corresponded to deprivation experiences between T3 and T4)?

The DIPCare-Q index was completed by participants at both T3 and T4. We chose to use the DIPCare-Q scores from T3 as they would represent “baseline” relative deprivation experienced by students, rather than the evolution of deprivation experienced overtime.

4.2.3. It is unclear why year in school was not included as a specific predictor or covariate,

particularly given that school year (i.e., preclinical vs. clinical phase) has been identified as a significant predictor of medical student wellbeing in multiple studies.

Thank you for this valuable feedback. We would like to confirm that students' year of study was included as a covariate in analyses, as described in subsection 2.3.1 (Sociodemographic Characteristics). We agree that school year has been identified as a predictor of student mental wellbeing in previous studies. However, the primary aim of our study was to examine the predictive value of overall deprivation, operationalized through a composite score encompassing multiple sociodemographic factors. As such, year of study was included as an adjustment variable rather than as a primary predictor.
In addition, the longitudinal design of our study imposed certain constraints. First-year students were not eligible for participation at T4 to prioritize longitudinal follow-up, and sixth-year students at T3 could not be followed up due to graduation. These limitations resulted in an incomplete overlap of study years across time points, thereby restricting our ability to conduct robust comparisons between preclinical and clinical stages.

We have clarified this point in the revised manuscript on page 10:

Preclinical and clinical stages were not included as a measure as first-year students were not eligible for participation at T4 to prioritize longitudinal follow up, and sixth-year students at T3 could not be followed up due to graduation.

5. Results:
5.1. The calculations of response rates are confusing. For example, 1101 respondents out of 1941 eligible participants would be 56.7%; 849 out of 1267 eligible participants would be 67%.
Thank you for noticing this statistical error. Indeed, the correct response rates have been revised and amended in the manuscript as follows (page 13):

At T3, 1101 (56.7% response rate) eligible students completed the survey; 35 were excluded for failure of attention questions or for technical issues. Analyses required the exclusion of sixth year students (n=118), as they would be ineligible for participation at T4, leading to a final sample of 948 students. At T4, first year students were ineligible to participate due to funding restrictions and were chosen for exclusion as their participation would not provide longitudinal data. Among eligible students (n= 1267), 849 (67% response rate) completed the survey. The total sample resulted in 819 students after exclusion of questionnaires for failure of attention questions or incoherent answers.

For further clarity, we have also included a flow chart of student participation in our supplemental files.

5.2. The text-based description of how many individuals participated is inconsistent with the information in supplemental Table 1. Please clarify.

Thank you for this comment. The information provided in supplemental Table 1 solely regards independent t-tests and chi-square tests conducted to compare students having participated at both T3 and T4 (n=631) and students having solely participated at T3 (n=317). We have reviewed this table for statistical errors and have modified the paragraph structure to better distinguish the participation calculation from these studies (page 13).

5.3. It may be preferable to use a different term (other than “single participants”) to describe individuals who only provided data at one timepoint, in order to prevent confusion with unmarried participants.

We would like to thank the reviewer for this insightful comment. We have modified the term “single participant” to “single-timepoint participant” where applicable across the revised manuscript and its supplemental material.

5.4. Do the authors have any hypotheses/explanation for why participants in their first year were less likely to participate at both time points? Were the single timepoint participants the individuals who were not eligible to participate at T4?
Thank you for this interesting query. A possible hypothesis could be the mental load that the first year of medical school represents to students. Indeed, it is a challenging and selective year for students, many of whom are entering the academic environment for the first time. It may be reasonable to suggest that faced with a rigorous programme and a competitive atmosphere, student participation may be hindered at this stage in the curriculum. Single-timepoint participants are individuals who were eligible to participate at both T3 and T4 but dropped-out. This latter point has been clarified in the method (page 13).

5.5. In section 3.2, the results seem to reflect only the double timepoint participants—for example, n=84 students are described as being in their first year, but in the supplemental table 1, it indicates that n=190 students participated. Please clarify.

We thank the reviewer for this helpful comment. Section 3.2 presents the descriptive statistics of measures at T3 for our central study population of students who completed both T3 and T4, while Supplemental Table 1 reports the attrition studies conducted between with these students with those who completed only T3. Indeed, the description in this section may have led to some confusion. The primary focus of this study is on longitudinal data from the double participant group (N=631). To distinguish these primary analyses from the attrition analyses, we have modified the Sample (page 13) and descriptive analyses (page 14) subsections as follows:

Sample (page 13):
A final sample of N=948 students participated at T3. Of these, 631 (66.6%) also participated at T4 while 317 (33.4%) did not, corresponding to an attrition rate of 33.4%. The final sample for longitudinal analyses therefore comprised of 631 students who participated at both T3 and T4.
Independent t-tests and chi-square tests were conducted to compare participants who completed both T3 and T4 with those who dropped out after T3.

Descriptive analyses (page 14):
Among the 631 double participants at T3, 67.5% participants identified themselves as female, 32% as male and 0.5% as non-binary.

5.6. Please consider conducting analyses to assess whether there is a significant association between year or preclinical/clinical phase of school and wellbeing.
We would like to thank the reviewer for this valuable suggestion. As stated in response to your previous comment (4.2.3.), year of study was included as a covariate in longitudinal

analyses. As previously mentioned, the central aim of this study was to explore the predictive value of living in a context of deprivation on mental wellbeing in medical students. While you raise a pertinent point on the importance of study stage on wellbeing, this analysis surpasses our central goal and is limited due to the exclusion of first-year students at T4 and sixth-year students at T3. Furthermore, the ETMED-L study team has already analysed our data and published the outcomes of mental health during the course of the medical curriculum (Carrard V, Berney S, Bourquin C, Ranjbar S, Castelao E, Schlegel K, et al. (2024) Mental health and burnout during medical school: Longitudinal evolution and covariates. PLoS ONE 19(4): e0295100. https://doi.org/10.1371/journal.pone.0295100).

5.7. My primary concerns relate to the longitudinal analyses—for step 4, please explain the rationale for considering deprivation only at T3, and not T4 (or not change in deprivation from T3 to T4) when predicting mental health at T4. For steps 5 and 6, please clarify whether deprivation at T3 or T4 is included in the analysis.
We thank you for sharing this valuable comment. Our primary aim was to test the prospective (cross-lagged) association between deprivation and subsequent mental health. For this reason, we did not include deprivation at T4 as a predictor of mental health at T4, as this would not preserve temporal precedence and could introduce issues of reverse causation. Similarly, modelling change in deprivation between T3 and T4 was beyond the scope of the present study, which focused specifically on prospective associations.

For Steps 5 and 6, deprivation measured at T3 was consistently used as the predictor variable, in line with this longitudinal framework. To improve clarity, we have modified the description of these analyses to highlight the use of T3 deprivation scores as predictors of mental health at T4 for Step 4 through 6 as follows (page 12):

Hence, hierarchical linear regression models were conducted in six steps to assess associations between baseline deprivation at T3 and dependent variables (i.e. depression, anxiety, suicidal ideation and burnout scores) both cross-sectionally and longitudinally.(…) Steps 4 through 6 assessed the prospective association between deprivation scores at T3 and mental health outcomes at T4.

We have also modified manuscript abstract (page 4) and subsection 3.3.2.(page 17) to further specify this.

5.8. Consider conducting additional analyses to explore whether change in deprivation status is associated with change in wellbeing measures. Given that the DIPCare-Q assesses deprivation in the recent past, it may be a better measure to assess current wellbeing. (It would be preferable to control for year in school or at least preclinical vs. clinical phase when conducting these analyses.)

Thank you for this thoughtful suggestion. We agree that examining whether changes in deprivation are related to changes in wellbeing represents an important and complementary research question. However, our primary study objective was to assess prospective associations (deprivation at T3 predicting student wellbeing at T4), rather than modelling students' change in deprivation. Accordingly, our analyses aimed to preserve a clear temporal ordering between exposure and outcome. Analyses based on change scores address a different research question e.g. whether changes in deprivation are associated with changes in wellbeing, which may be more prone to measurement error. Therefore, we focused on the temporally ordered exposure (baseline deprivation at T3) to avoid concurrent specification at

T4. Future studies could explore dynamic changes in deprivation and their relationship with wellbeing over time. We have clarified this rationale in the Methods and have added this latter point to our discussion section for next steps:

Methods (page 12):
Our study focused on the prospective association between deprivation and mental health outcomes rather than modelling deprivation score changes and their association with changes in wellbeing.

Furthering research (page 23):
Finally, future studies could explore dynamic changes in deprivation and their relationship with wellbeing over time.

As mentioned in our response 5.6., while our study used year of study as a covariate in analyses, our ETMED-L colleagues have specifically investigated the longitudinal association between curriculum year and wellbeing outcomes. As suggested, the time frames used by certain measures limits the accuracy of results; future studies should aim to use measures with similar time frames to best reflect the associations analysed.

6. Discussion
6.1. The Discussion will likely require significant revision after edits are made to the results section.
As suggested, we have modified the discussion to best reflect the previous revisions made in response to editors' and reviewers' comments.

6.2. The discussion appears to draw conclusions and make assertions that extend beyond the study results. Please temper these assertions to ensure accuracy.

Thank you for this valuable comment. To address this comment, we have revised the discussion (page 20-24), conclusion (page 24) as well as the abstract (page 4) accordingly to avoid any conclusions not supported by our results.

6.3. Additional limitations include use of self-report data and associated risks for biased reporting, unclear generalizability give the confusing response rates, variability in time frame for the deprivation questions (making it difficult to draw conclusions about direct impact, lack of information about deprivation experiences in early life, unequal representation of class years in the sample.

Thank you to the reviewer for this final comment. In response to the overall suggestions made by reviewers and editors, we have modified the Limitations subsection (page 23) in response:

Response bias may have arisen due to inconsistent use of time frames across mental health, deprivation and protective factor measures. Longer or unspecified time frames may have led to under-reporting of symptoms, while shorter time frames may have caused over-reporting, thus affecting the accuracy of the findings. Switzerland has a relatively high economic standing; extrapolating our findings to countries with different economic contexts may be challenging. Additionally, our participants may have differing perceptions of socio-economic status given their geopolitical context, further limiting the generalisability of our findings.

VERSION 2 - REVIEW

**Reviewer**	**2**
**Name**	**Popescu, Codruta**
**Affiliation**	**Universitatea de Medicina si Farmacie Iuliu Hatieganu**
**Date**	**16-Apr-2026**
**COI**	

In my opinion the authors have answered to all reviewers comment, they have modify the paper and improved it. In the present form the paper is ready for publication and it should be accepted.
