# Supplementary material for: Mental health and burnout among Swiss medical students living in contexts of deprivation: a longitudinal study
Source: BMJ Open. 2026 Jul 29;16(7):e114025. doi: 10.1136/bmjopen-2025-114025 (PMC13435915; doi:10.1136/bmjopen-2025-114025)
Supplement: Supplementary file 3 [file bmjopen-16-7-s002.docx]

12. **SUPPLEMENTAL INFORMATION**

**S1 Table**

| *Frequencies and Chi-square results for sex, nationality and curriculum year among double (n=631) and single-timepoint participants (n=317).* | | | | | | | | |
| --- | --- | --- | --- | --- | --- | --- | --- | --- |
|  | Double participants | | Single-timepoint participants | |  |  |  |  |
| Variables | N | % | N | % | χ ^2^ | *p-value* | *phi* | *p-value* |
| Gender |  |  |  |  | 0.75 | 0.39 | 0.03 | 0.35 |
| Male | 202 | 32.0 | 92 | 29.0 | — |  | — |  |
| Female & N-B* | 429 | 68.0 | 225 | 71.0 | — |  | — |  |
| Nationality |  |  |  |  | 16.78^*^ | <0.001 | 0.14^*^ | <0.001 |
| Swiss† | 497 | 78.8 | 210 | 66.2 | — |  | — |  |
| Other | 134 | 21.2 | 107 | 33.8 | — |  | — |  |
| Curriculum year |  |  |  |  | 237.62^*^ | <0.001 | 0.50^*^ | <0.001 |
| Year 1 | 84 | 13.3 | 190 | 59.9 | — |  | — |  |
| Year 2 | 131 | 20.8 | 21 | 6.6 | — |  | — |  |
| Year 3 | 149 | 23.6 | 37 | 11.7 | — |  | — |  |
| Year 4 | 143 | 22.7 | 18 | 5.7 | — |  | — |  |
| Year 5 | 124 | 19.7 | 51 | 16.1 | — |  | — |  |

*Note.* *NB= non-binary, †Swiss if mother tongue= Swiss-French, Swiss-German or Swiss-Italian.

**S2 Table**

| *Descriptives of independent t-test comparing double participants (n= 631) and single-timepoint participants (n=317) at T3.* | | | | | | | |
| --- | --- | --- | --- | --- | --- | --- | --- |
|  | Double participants | | Single-timepoint participants | |  |  |  |
| Variables | M | SD | M | SD | t(*df*) | *p-value* | Cohen’s *d* |
| Age | 22.11 | 2.95 | 20.81 | 2.87 | 6.44 (942) | <0.001 | 0.44 |
| ODI^a^ | 0.69 | 0.81 | 0.81 | 0.88 | -2.03 (944) | 0.04 | -0.14 |
| CESD^b^ | 17.93 | 10.35 | 22.29 | 12.43 | -5.33 (523.77) | <0.001 | -0.39 |
| STAI^c^ | 45.59 | 11.24 | 48.94 | 12.02 | -4.19 (936) | <0.001 | -0.29 |
| BDI^d^ | 0.59 | 1.01 | 0.90 | 1.16 | -3.95 (545.64) | <0.001 | -0.29 |
| MBI-Ex^e^ | 16.66 | 4.96 | 17.13 | 5.23 | -1.36 (935) | 0.17 | -0.09 |
| MBI-Cy^f^ | 9.16 | 4.22 | 9.51 | 4.56 | -1.16 (571.97) | 0.25 | -0.08 |
| MBI-Ae^g^ | 24.38 | 4.34 | 23.47 | 4.53 | 2.99 (935) | 0.003 | 0.21 |
| Cop_E^h^ | 9.52 | 3.76 | 10.16 | 4.25 | -2.26 (551.50) | 0.02 | -0.16 |
| Cop_P^i^ | 7.22 | 1.73 | 7.17 | 1.79 | 0.37 (935) | 0.71 | 0.03 |
| Cop_H^j^ | 5.58 | 2.75 | 5.01 | 2.71 | 2.98 (934) | 0.003 | 0.21 |
| SocSup^k^ | 8.19 | 1.78 | 7.89 | 2.07 | 2.15 (535.18) | 0.03 | 0.16 |

*Note*. a.ODI=Overall deprivation index; b.CESD= depression; c.STAI= anxiety; d.BDI= suicidal ideation; e.MBI-Ex= emotional exhaustion, f.MBI-Cy= cynicism, g.MBI-Ae= academic efficacy; h.Cop_E= emotion-focused coping; i.Cop_P= problem-focused coping; j.Cop_H= help-seeking coping; k.SocSup= social support. *p*=two-tailed. Cohen’s *d* effect size was interpreted as follows: 0.2-0.49= small effect; 0.5-0.79=medium effect, 0.8=large effect.

**Table S3**

| *Cross-sectional Hierarchical Linear Regression Analyses Testing the Association Between Overall Deprivation and Depression, Anxiety and Suicidal Ideation Scores (N=631).* | | | | | | | | | | | | | | | |
| --- | --- | --- | --- | --- | --- | --- | --- | --- | --- | --- | --- | --- | --- | --- | --- |
|  | Step 1 | | | | | | Step 2 | | | | | Step 3 | | | |
|  | B | SE B | | *β* | | *p* | B | SE B | *β* | | *p* | B | SE B | *β* | *p* |
| Depression (CESD) | | | | | | | | | | | | | | | |
| ODI* | 4.16 | 0.48 | 0.33 | | <0.001 | | 4.32 | 0.49 | 0.34 | <0.001 | | 3.00 | 0.43 | 0.23 | <0.001 |
| Cop_E† | — | — | — | | — | | — | — | — | — | | 1.15 | 0.09 | 0.42 | <0.001 |
| Cop_P‡ | — | — | — | | — | | — | — | — | — | | 0.03 | 0.19 | 0.005 | 0.87 |
| Cop_H§ | — | — | — | | — | | — | — | — | — | | -0.340 | 0.13 | -0.09 | 0.01 |
| SocSup‖ | — | — | — | | — | | — | — | — | — | | -0.99 | 0.20 | -0.17 | <0.001 |
| R² | — | 0.11 | — | | — | | — | 0.18 | — |  | | — | 0.39 | — | — |
| F (*df*) | — | 74.89 (1,627) | — | | <0.001 | | — | 26.80 (5,620) | — | <0.001 | | — | 43.54 (9,615) | — | <0.001 |
| ∆R² | — | — | — | | — | | — | 0.10 | — |  | | — | 0.05 | — | — |
| F change(*df*) | — | — | — | | — | | — | 78.51(1,620) | — | <0.001 | | — | 47.98 (1,615) | — | <0.001 |
| Anxiety (STAI) | | | | | | | | | | | | | | | |
| ODI* | 3.88 | 0.53 | 0.28 | | <0.001 | | 4.16 | 0.53 | 0.30 | <0.001 | | 2.53 | 0.44 | 0.18 | <0.001 |
| Cop_E† | — | — | — | | — | | — | — | — | — | | 1.53 | 0.10 | 0.51 | <0.001 |
| Cop_P‡ | — | — | — | | — | | — | — | — | — | | -0.18 | 0.20 | -0.03 | 0.35 |
| Cop_H§ | — | — | — | | — | | — | — | — | — | | -0.31 | 0.13 | -0.08 | 0.02 |
| SocSup‖ | — | — | — | | — | | — | — | — | — | | -1.13 | 0.21 | -0.18 | <0.001 |
| R² | — | 0.08 | — | | — | | — | 0.17 | — | — | | — | 0.47 | — | — |
| F (*df*) | — | 53.44 (1,627) | — | | <0.001 | | — | 26.18 (5,620) | — | <0.001 | | — | 59.75 (9,615) | — | <0.001 |
| ∆R² | — | — | — | | — | | — | 0.08 | — |  | | — | 0.03 | — | — |
| F change(*df*) | — | — | — | | — | | — | 61.73 (1,620) | — | <0.001 | | — | 33.07 (1,615) | — | <0.001 |
| Suicidal Ideation (BDI) | | | | | | | | | | | | | | | |
| ODI* | 0.34 | 0.05 | 0.27 | | <0.001 | | 0.34 | 0.05 | 0.27 | <0.001 | | 0.26 | 0.05 | 0.21 | <0.001 |
| Cop_E† | — | — | — | | — | | — | — | — | — | | 0.07 | 0.01 | 0.28 | <0.001 |
| Cop_P‡ | — | — | — | | — | | — | — | — | — | | -0.03 | 0.02 | -0.06 | 0.11 |
| Cop_H§ | — | — | — | | — | | — | — | — | — | | -0.06 | 0.01 | -0.16 | <0.001 |
| SocSup‖ | — | — | — | | — | | — | — | — | — | | -0.05 | 0.02 | -0.08 | 0.04 |
| R² | — | 0.07 | — | | — | | — | 0.08 | — | — | | — | 0.20 | — | — |
| F (*df*) | — | 49.98 (1,627) | — | | <0.001 | | — | 10.68 (5,620) | — | <0.001 | | — | 16.81 (9,615) | — | <0.001 |
| ∆R² | — | — | — | | — | | — | 0.07 | — | — | | — | 0.04 | — | — |
| F change(*df*) | — | — | — | | — | | — | 46.25 (1,620) | — | <0.001 | | — | 29.63 (1,615) | — | <0.001 |
| Burnout | | | | | | | | | | | | | | | |
| Emotional Exhaustion (MBI_Ex) | | | | | | | | | | | | | | | |
| ODI* | 1.55 | 0.24 | 0.25 | | <0.001 | | 1.72 | 0.24 | 0.28 | <0.001 | | 1.30 | 0.24 | 0.21 | <0.001 |
| Cop_E† | — | — | — | | — | | — | — | — | — | | 0.39 | 0.05 | 0.30 | <0.001 |
| Cop_P‡ | — | — | — | | — | | — | — | — | — | | -0.01 | 0.10 | -0.003 | 0.93 |
| Cop_H§ | — | — | — | | — | | — | — | — | — | | -0.13 | 0.07 | -0.07 | 0.07 |
| SocSup‖ | — | — | — | | — | | — | — | — | — | | -0.23 | 0.11 | -0.08 | 0.04 |
| R² | — | 0.06 | — | | — | | — | 0.11 | — | — | | — | 0.21 | — | — |
| F (*df*) | — | 43.12 (1,626) | — | | <0.001 | | — | 15.77 (5,619) | — | <0.001 | | — | 18.18 (9,615) | — | <0.001 |
| ∆R² | — | — | — | | — | | — | 0.07 | — | — | | — | 0.04 | — | — |
| F change(*df*) | — | — | — | | — | | — | 50.37 (1,619) | — | <0.001 | | — | 30.55 (1,615) | — | <0.001 |
| Cynicism (MBI_Cy) | | | | | | | | | | | | | | | |
| ODI* | 0.61 | 0.21 | 0.12 | | 0.003 | | 0.74 | 0.21 | 0.14 | <0.001 | | 0.47 | 0.21 | 0.09 | 0.03 |
| Cop_E† | — | — | — | | — | | — | — | — | — | | 0.27 | 0.05 | 0.24 | <0.001 |
| Cop_P‡ | — | — | — | | — | | — | — | — | — | | -0.18 | 0.09 | -0.07 | 0.06 |
| Cop_H§ | — | — | — | | — | | — | — | — | — | | -0.09 | 0.06 | -0.06 | 0.16 |
| SocSup‖ | — | — | — | | — | | — | — | — | — | | -0.21 | 0.10 | -0.09 | 0.03 |
| R² | — | 0.01 | — | | — | | — | 0.03 | — | — | | — | 0.11 | — | — |
| F(*df*) | — | 8.73 (1,626) | — | | 0.003 | | — | 4.34 (5,619) | — | <0.001 | | — | 8.64 (9,615) | — | <0.001 |
| ∆R² | — | — | — | | — | | — | 0.02 | — | — | | — | 0.01 | — | — |
| F change(*df*) | — | — | — | | — | | — | 11.84 (1,619) | — | <0.001 | | — | 4.84 (1,615) | — | 0.03 |
| Academic Efficacy (MBI_Ae) | | | | | | | | | | | | | | | |
| ODI* | -0.61 | 0.21 | -0.11 | | 0.005 | | -0.59 | 0.22 | -0.11 | 0.009 | | -0.24 | 0.20 | -0.04 | 0.23 |
| Cop_E† | — | — | — | | — | | — | — | — | — | | -0.32 | 0.04 | -0.28 | <0.001 |
| Cop_P‡ | — | — | — | | — | | — | — | — | — | | 0.61 | 0.09 | 0.24 | <0.001 |
| Cop_H§ | — | — | — | | — | | — | — | — | — | | 0.22 | 0.06 | 0.14 | <0.001 |
| SocSup‖ | — | — | — | | — | | — | — | — | — | | 0.51 | 0.09 | 0.21 | <0.001 |
| R² | — | 0.01 | — | | — | | — | 0.01 | — | — | | — | 0.25 | — | — |
| F(*df*) | — | 8.12 (1,626) | — | | 0.005 | | — | 1.67 (5,619) | — | 0.14 | | — | 23.27 (9,615) | — | <0.001 |
| ∆R² | — | -- | — | | — | | — | 0.01 | — | — | | — | 0.002 | — | — |
| F change(*df*) | — | -- | — | | — | | — | 6.87 (1,619) | — | 0.009 | | — | 1.44 (1,615) | — | 0.23 |

*Note*. *ODI= Overall deprivation index, †Cop_E= emotion focused coping score, ‡Cop_P= problem-focused coping score, §Cop_H= help-seeking coping score, ‖SocSup= social support score.

**Table S4**

| *Longitudinal Hierarchical Linear Regression Analyses Testing the Association Between Overall Deprivation and Depression, Anxiety and Suicidal Ideation Scores (N=631).* | | | | | | | | | | | | |
| --- | --- | --- | --- | --- | --- | --- | --- | --- | --- | --- | --- | --- |
|  | Step 4 | | | | Step 5 | | | | Step 6 | | | |
|  | B | SE B | *β* | *p* | B | SE B | *β* | *p* | B | SE B | *β* | *p* |
| Depression symptoms (CESD) | | | | | | | | | | | | |
| ODI* | 4.34 | 0.52 | 0.32 | <0.001 | 1.90 | 0.46 | 0.14 | <0.001 | 1.85 | 0.46 | 0.14 | <0.001 |
| CESD-T3† | — | — | — | — | 0.61 | 0.04 | 0.57 | <0.001 | 0.51 | 0.04 | 0.47 | <0.001 |
| Cop_E‡ | — | — | — | — | — | — | — | — | 0.46 | 0.11 | 0.15 | <0.001 |
| Cop_P§ | — | — | — | — | — | — | — | — | -0.13 | 0.19 | -0.02 | 0.52 |
| Cop_H‖ | — | — | — | — | — | — | — | — | -0.15 | 0.14 | -0.04 | 0.26 |
| SocSup¶ | — | — | — | — | — | — | — | — | -0.42 | 0.21 | -0.07 | 0.05 |
| R² | — | 0.10 | — | — | — | 0.42 | — | — | — | 0.45 | — | — |
| F (*df*) | — | 70.42 (1,624) | — | <0.001 | — | 75.20 (6,616) | — | <0.001 | — | 49.35 (10,612) | — | <0.001 |
| ∆R² | — | — | — | — | — | 0.02 | — | — | — | 0.02 | — | — |
| F change(*df*) | — | — | — | — | — | 16.80 (1,616) | — | <0.001 | — | 16.28 (1,612) | — | <0.001 |
| Anxiety (STAI) | | | | | | | | | | | | |
| ODI* | 3.72 | 0.55 | 0.26 | <0.001 | 0.82 | 0.41 | 0.06 | 0.04 | 0.82 | 0.41 | 0.06 | 0.04 |
| STAI-T3† | — | — | — | — | 0.76 | 0.03 | 0.73 | <0.001 | 0.71 | 0.04 | 0.69 | <0.001 |
| Cop_E‡ | — | — | — | — | — | — | — | — | 0.18 | 0.11 | 0.06 | 0.09 |
| Cop_P§ | — | — | — | — | — | — | — | — | -0.17 | 0.18 | -0.03 | 0.33 |
| Cop_H‖ | — | — | — | — | — | — | — | — | -0.001 | 0.12 | 0.00 | 0.99 |
| SocSup¶ | — | — | — | — | — | — | — | — | -0.30 | 0.19 | -0.05 | 0.12 |
| R² | — | 0.07 | — | — | — | 0.58 | — | — | — | 0.59 | — | — |
| F (*df*) | — | 44.85 (1,624) | — | <0.001 | — | 142.95 (6,616) | — | <0.001 | — | 86.82 (10,612) | — | <0.001 |
| ∆R² | — | — | — | — | — | 0.003 | — | — | — | 0.003 | — | — |
| F change(*df*) | — | — | — | — | — | 3.99 (1,616) | — | 0.04 | — | 3.99 (1,612) | — | 0.04 |
| Suicidal Ideation (BDI) | | | | | | | | | | | | |
| ODI* | 0.26 | 0.05 | 0.21 | <0.001 | 0.11 | 0.05 | 0.09 | 0.02 | 0.08 | 0.05 | 0.07 | 0.08 |
| BDI-T3† | — | — | — | — | 0.43 | 0.04 | 0.44 | <0.001 | 0.37 | 0.04 | 0.38 | <0.001 |
| Cop_E‡ | — | — | — | — | — | — | — | — | 0.03 | 0.01 | 0.11 | 0.005 |
| Cop_P§ | — | — | — | — | — | — | — | — | -0.004 | 0.02 | -0.01 | 0.83 |
| Cop_H‖ | — | — | — | — | — | — | — | — | -0.01 | 0.01 | -0.03 | 0.46 |
| SocSup¶ | — | — | — | — | — | — | — | — | -0.06 | 0.02 | -0.12 | 0.003 |
| R² | — | 0.04 | — |  | — | 0.23 | — | — | — | 0.26 | — | — |
| F(*df*) | — | 29.52 (1,624) | — | <0.001 | — | 30.97 (6,616) | — | <0.001 | — | 21.40 (10,612) | — | <0.001 |
| ∆R² | — | — | — | — | — | 0.01 | — | — | — | 0.004 | — | — |
| F change(*df*) | — | — | — | — | — | 5.12 (1,616) | — | 0.02 | — | 3.02 (1,612) | — | 0.08 |
| Burnout | | | | | | | | | | | | |
| Emotional Exhaustion (MBI_Ex) | | | | | | | | | | | | |
| ODI* | 1.13 | 0.24 | 0.19 | <0.001 | 0.24 | 0.19 | 0.04 | 0.22 | 0.19 | 0.19 | 0.03 | 0.34 |
| MBI_Ex-T3† | — | — | — | — | 0.61 | 0.03 | 0.62 | <0.001 | 0.58 | 0.03 | 0.59 | <0.001 |
| Cop_E‡ | — | — | — | — | — | — | — | — | 0.10 | 0.04 | 0.08 | 0.02 |
| Cop_P§ | — | — | — | — | — | — | — | — | -0.09 | 0.08 | -0.03 | 0.28 |
| Cop_H‖ | — | — | — | — | — | — | — | — | -0.13 | 0.06 | -0.07 | 0.02 |
| SocSup¶ | — | — | — | — | — | — | — | — | -0.10 | 0.09 | -0.04 | 0.23 |
| R² | — | 0.04 | — | — | — | 0.46 | — | — | — | 0.47 | — | — |
| F (*df*) | — | 23.04 (1,624) | — | <0.001 | — | 86.65 (6,616) | — | <0.001 | — | 54.84 (10,612) | — | <0.001 |
| ∆R² | — | — | — | — | — | 0.001 | — | — | — | 0.001 | — | — |
| F change(*df*) | — | — | — | — | — | 1.48 (1,616) | — | 0.22 | — | 0.92 (1,612) | — | 0.34 |
| Cynicism (MBI_Cy) | | | | | | | | | | | | |
| ODI* | 0.51 | 0.21 | 0.09 | 0.02 | 0.18 | 0.18 | 0.03 | 0.31 | 0.13 | 0.18 | 0.02 | 0.49 |
| MBI_Cy-T3† | — | — | — | — | 0.57 | 0.03 | 0.56 | <0.001 | 0.54 | 0.03 | 0.53 | <0.001 |
| Cop_E‡ | — | — | — | — | — | — | — | — | 0.07 | 0.04 | 0.06 | 0.12 |
| Cop_P§ | — | — | — | — | — | — | — | — | -0.13 | 0.08 | -0.05 | 0.11 |
| Cop_H‖ | — | — | — | — | — | — | — | — | -0.10 | 0.06 | -0.06 | 0.08 |
| SocSup¶ | — | — | — | — | — | — | — | — | -0.13 | 0.09 | -0.05 | 0.14 |
| R² | — | 0.01 | — | — | — | 0.33 | — | — | — | 0.34 | — | — |
| F (*df*) | — | 5.89 (1,624) | — | 0.02 | — | 50.40 (6,616) | — | <0.001 | — | 32.24 (10,612) | — | <0.001 |
| ∆R² | — | — | — | — | — | 0.001 | — | — | — | 0.00 | — | — |
| F change(*df*) | — | — | — | — | — | 1.02 (1,616) | — | 0.31 | — | 0.46 (1,612) | — | 0.49 |
| Academic Efficacy (MBI_Ae) | | | | | | | | | | | | |
| ODI* | -0.28 | 0.22 | -0.05 | 0.19 | 0.14 | 0.17 | 0.03 | 0.41 | 0.28 | 0.17 | 0.05 | 0.10 |
| MBI_Ae-T3† | — | — | — | — | 0.68 | 0.03 | 0.67 | <0.001 | 0.64 | 0.03 | 0.63 | <0.001 |
| Cop_E‡ | — | — | — | — | — | — | — | — | -0.13 | 0.04 | -0.11 | 0.001 |
| Cop_P§ | — | — | — | — | — | — | — | — | -0.08 | 0.08 | -0.03 | 0.34 |
| Cop_H‖ | — | — | — | — | — | — | — | — | 0.01 | 0.05 | 0.004 | 0.92 |
| SocSup¶ | — | — | — | — | — | — | — | — | 0.15 | 0.08 | 0.06 | 0.07 |
| R² | — | 0.003 | — | — | — | 0.46 | — | — | — | 0.47 | — | — |
| F(*df*) | — | 1.67 (1,624) | — | 0.19 | — | 86.06 (6,616) | — | <0.001 | — | 54.31 (10,612) | — | <0.001 |
| ∆R² | — | — | — | — | — | 0.001 | — | — | — | 0.002 | — | — |
| F change(*df*) | — | — | — | — | — | 0.68 (1,616) | — | 0.41 | — | 2.68 (1,612) | — | 0.10 |

*Note*. *ODI= Overall deprivation index, †Score name-T3= Baseline mental health score ‡Cop_E= emotion focused coping score, §Cop_P= problem-focused coping score, ‖Cop_H= help-seeking coping score, ¶SocSup= social support score.

**APPENDIX A**

**A.1 DIPCare-Q overall index calculation & item examples**

Overall deprivation index= [material deprivation] × 0.810 + [social deprivation] × 0.455 + [health deprivation] × 0.711

Material item: “Over the last 12 months, have you had trouble paying your household bills (e.g. tax, insurance, phone, electricity, credit cards, etc.)?”

Social item: “In the last 3 months, have you spent an evening with family or friends?”

Health item: “Do you currently suffer from a physical disability that has a major impact on your daily life?”

**A.2 Beck Depression Inventory item responses**

- Item 1: 0=I’m not particularly discouraged by the future, 1=I feel discouraged by the future, 2=I feel that I have no expectations in life, 3=I feel that the future is hopeless, and things can’t improve.
- Item 2: 0=I don't have any thoughts of killing myself; 1=I have thoughts of killing myself, but I would not carry them out; 2=I would like to kill myself; 3=I would kill myself if I had the chance.

**A.3 Social support item responses**

- Item 1 (practical support): If necessary, in your opinion, to what extent can your partner provide you with practical help, this means concrete help or useful advice, if 0 means "not at all" and 10 "a great deal"?
- Item 2 (emotional support): To what extent can your someone be available in case of need and show understanding, by talking with you for example, if 0 means "not at all" and 10 "a great deal"?
